# A universal CRISPR-Cas14a responsive triple-sensitized upconversion photoelectrochemical sensor

**DOI:** 10.1186/s12951-023-02163-z

**Published:** 2023-10-26

**Authors:** Yu Wang, Yuan Peng, Huanying Zhou, Zhixian Gao

**Affiliations:** Tianjin Key Laboratory of Risk Assessment and Control Technology for Environment and Food Safety, Tianjin Institute of Environmental and Operational Medicine, 300050 Tianjin, P.R. China

**Keywords:** CRISPR-Cas14a, Strand displacement amplification, Upconversion, Photoelectrochemical sensor, Near-infrared

## Abstract

It has recently been discovered that, like other members of the Cas family (12a and 13a), the clustered regularly interspaced short palindrome repeat CRISPR-Cas14a system not only mediates high-sensitivity detection with exceptionally strong gene editing ability but is also generally useful for DNA detection via fluorescence. Photoelectrochemical (PEC) sensors have been widely applied as efficient analytical tools. Measuring electrical signals is more cost-effective and the necessary equipment is more easily portable than fluorescence signal detectors, but their stability still needs to be improved. The high base resolution of CRISPR-Cas14a can compensate for such shortcomings. Therefore, electrical signals and fluorescence signals were combined, and the development of a universal CRISPR-Cas14a-responsive ultrasensitive upconversion PEC sensor is described in this paper. Moreover, strand displacement amplification (SDA) and a near-infrared (NIR) light source were utilized to further improve the stability and sensitivity of the photoelectric signals. At the same time, the modified working electrode (UCNPs-ssDNA-CdS@Au/ITO) on the three-electrode disposable sensor was used as the reporter probe, which cooperates with the trans-cleavage activity of Cas14a endonuclease. To verify the universality of this sensor, the UCNPs-Cas14a-based PEC sensor was applied for the detection of the small-molecule toxin T2 and protein kinase PTK7. Here, we report that the limit of detection of this reagent was within the fg range, successfully applied to the detection of T2 in oats and PTK7 in human serum. We propose that by combining PEC and CRISPR-14a, UCNPs-Cas14a-based PEC sensors could become powerful drivers for the extensive development of ultrasensitive, accurate and cost-effective universal sensors for detection and diagnosis.

**Supplementary Information:**

The online version contains supplementary material available at 10.1186/s12951-023-02163-z.

## Introduction

Recently, researchers have found that there is a Cas14a protein (Currently, Cas14 nuclease and other V-F effectors are grouped and systematically named Cas12f nuclease.) in the family of clustered regularly interspaced short palindrome repeats and their related proteins (CRISPR-Cas system), which is about half the size of other Cas proteins (40–70 kDa) [1–3]. Cas14a can bind to and cleave the target sequence of single-stranded DNA (ssDNA) [4]. Unlike Cas9, Cas14a requires no PAM sequence [5]. In addition to such cis-cleavage, Cas14a, similar to Cas13a and Cas12a, can indiscriminately trans-cleave ssDNA [6]. However, Cas14a is more specific than Cas13a or Cas12a in identifying ssDNA [7]. It is believed that Cas14a binds to ssDNA rather than double-stranded DNA, so it may not be a good genome-editing tool [8]. However, many researchers have developed SHERLOCK and DETECTR sensors using Cas12a and Cas13a to quickly detect pathogen sequences and the presence of genetic mutations [9, 10], while Cas14a-based detector sensors have been less frequently reported [11–13]. Therefore, here we describe techniques for combining Cas14a aptamers and isothermal amplification, with the aim of applying Cas14a not only to high-sensitivity sensing detection, but to extend the detection range, so as to identify a wide variety of targets from small molecules to proteins.

Currently, most sensors based on the CRISPR-Cas system use fluorescence as the output signal [14, 15]. However, using electrical signals is more cost-effective and requires equipment that is more easily portable than fluorescence signal detectors [16]. Recently, photoelectrochemistry (PEC), a new analytical tool combining photochemistry and electrochemistry, has been extensively developed. The main principle of this technology is that electron-hole pair separation and charge transfer on the photoactive material results in conversion to photoelectric signals on exposure to light. The corresponding photocurrent in this case is significantly affected by the electron donor/acceptor [17, 18]. The PEC-based biosensing platform is characterized by rapid signal reading, simple operation and high sensitivity [19, 20]. However, stability is one of the key factors limiting PEC biosensing [21, 22], but the stability of CRISPR-Cas14a in targeting nucleic acids can compensate for such shortcomings [23]. To further improve the stability and sensitivity of photoelectric signals, near-infrared (NIR) light with its low phototoxicity and background values can be used as the light source instead of the more traditional visible/ultraviolet light, exploiting upconversion nanoparticles (UCNPs) with light stability and long fluorescence lifetimes as the NIR light absorption medium [24, 25]. On this basis, the working principle of the electrode we designed (Figure S1) is to match the optical characteristics of UCNPs, Au and cadmium sulfide (CdS). When the electrode surface is exposed to 980 nm NIR light, UCNPs will be excited and emit 550 nm-wavelength light [26, 27]. This light emitted by the irradiated UCNPs transfers energy to Au through fluorescence resonance energy transfer (FRET) [28]. Au then transfers this accumulated energy to CdS through plasmon resonance energy transfer (PRET), producing electrons (e^−^) and photo-generated holes (h^+^) [29]. Because the excited electrons are transferred to the indium tin oxide (ITO) electrode, the photo-generated holes can be captured by the ascorbic acid (AA) in the solution [30]. The AA (C_6_H_8_O_6_) is oxidized to dehydroascorbic acid (C_6_H_6_O_6_), thereby effectively preventing the recombination of electrons and holes from enhancing the photocurrent.

On this basis, a universal CRISPR-Cas14a-responsive ultrasensitive upconversion PEC sensor is reported here. In this device, first, the non-nucleic acid targets are converted into DNA input sensors through the competitive binding of target and complementary strand to aptamer. A large amount of target ssDNA is thus obtained by strand displacement amplification (SDA) to amplify the signals. SDA is a method that uses DNA polymerase with strand-displacing activity to cleave the endonuclease in the primer to produce an initiation of amplification at the cut site. The incision site is regenerated in each round of amplification, thus triggering exponential amplification. The target ssDNA then triggers the cleavage activity of Cas14a in the PEC system, which maintains UCNPs at some distance from the electrode surface and prevent energy transfer to the Au on the electrode through FRET, thus weakening photoelectric signals. Additionally, an NIR PEC analytical method with high stability and sensitivity was explored through high-precision CRISPR-Cas14a and high-efficiency UCNPs, used for ultrasensitive detection of different types of targets. The CRISPR-Cas14a-responsive ultrasensitive upconversion PEC sensor has great potential application, for example, in the fields of foodstuff monitoring and clinical diagnosis.

## Experimental section

### Electrode modification

1. Pretreatment of ITO glass: The ITO glass was cut into rectangles (L: 4.0 cm, W: 0.6 cm), followed by ultrasonic cleaning with ammonia water (water: ammonia = 30:1), deionized water, absolute ethanol and sub-boiling water for 10 min.
2. Preparation of Au/ITO electrodes: The pretreated ITO was placed in 5.2 mL of plating solution (1.2 mL of 1 mM HAuCl_4_ + 4.0 mL of buffer solution, pH = 7.0), and Au nanoparticles were deposited *via* cyclic voltammetry. Deposition conditions: temperature: 30℃, potential: 0.1 to -0.9 V, scan rate: 0.05 V/s, number of scan cycles: 20, with nitrogen protection.
3. Preparation of CdS/ITO electrodes: 2 mL of 0.1 M CdS and 2.0 mL of 0.02 M Na_2_S_2_O_3_ solution were used as the electrolyte, and the pH was adjusted to 2–3 with 0.1 M HCl. CdS nanoparticles were deposited on the surface of ITO glass *via* cyclic voltammetry. Electrolysis parameters: deposition potential: -0.2 to -0.8 V, scan rate: 0.05 V/s, number of deposition cycles: 40, temperature: 50℃.
4. Preparation of CdS-Au/ITO electrodes: The CdS/ITO electrode prepared in (3) was put into the Au/ITO preparation solution, with the same deposition conditions as those for the preparation of Au/ITO electrode.
5. Preparation of CdS-Au-UCNPs/ITO electrodes: 200 µL of 2 mg/mL sulfhydryl-modified DNA-UCNPs were mixed with 5 µL of 26.7 mM TCEP, followed by reduction at room temperature for 30 min. This could then be directly mixed with CdS-Au/ITO electrode and allowed to react at room temperature for 2 h.

### PEC detection methods

**I. Magnetic bead recognition**:

1. 100 µL of magnetic beads were placed in a new centrifuge tube. The centrifuge tube was placed on a magnetic separator and left to stand for 1 min (hereinafter referred to as magnetic separation), and the supernatant was aspirated with a pipette.
2. The magnetic beads were washed ×3 with 1 mL of Buffer I.
3. 500 µL of biotinylated nucleic acid diluted with Buffer I (final concentration of DNA: 2 nmol/mL, that is, 100 µL of 10 µM DNA added with 400 µL of Buffer I) was added, and the mixture was vigorously shaken to resuspend the magnetic beads. The centrifuge tube was placed on a rotary mixer, and the mixture was rotated at room temperature for 30 min.
4. After magnetic separation, the supernatant was transferred into a new centrifuge tube.
5. The magnetic beads were washed ×3 according to the method in “2”).
6. According to the requirements of subsequent tests, an appropriate amount of low-salt buffer was added to resuspend the magnetic beads. After this step, the magnetic beads bound to biotinylated nucleic acid successfully, and could be used for subsequent operations.
7. MMPs-apt (500 µL, 1 mg/mL) and excess complementary oligonucleotide (cDNA) solution were hybridized at 37 °C for 30 min to obtain MMPs-apt-cDNA, and unreacted cDNA was removed by magnetic separation. Next the material was resuspended in 50 µL. Then 10 µL of the target (T2/PTK7) standard solution (1 pg/mL) at different concentrations was mixed with 10 µL of MMPs-apt-cDNA solution, followed by incubation at 37 °C for 1 h. Finally, the dissociated cDNA (11 µL) was collected by magnetic separation, and transferred to the SDA system.

**II. SDA isothermal amplification**:

1. The system volume was set to 20 µL: First, 11 µL of TarDNA and 2 µL of TemDNA (1 µmol/L) were added, heated in hot water bath at 95℃ for 5 min, and then slowly cooled.
2. 2 µL of CutSmart (10×) buffer, 3 µL of dNTP (10 mmol/L), and 1 µL of Klenow Fragment (3’-5’ exo-) (5,000 U/mL) were added and allowed to react for 15 min. Then 1 µL of Nt.BsmAI enzyme (10,000 U/mL) was added, mixed well and incubated at 37 °C for 3 h. Finally, the mixture was heated in hot water bath at 80℃ for 10 min to inactivate the enzyme.

**III. Reagent preparation**:

10× lysis buffer: 250 mM NaCl, 200 mM HEPES, pH 7.5, 10 mM DTT, 50% glycerol and 50 mM MgCl_2_.

Special electrolytes: 0.1 M PBS (pH 7.41), 0.01 M AA.

**IV. Cleavage steps**:

First, Cas14a (0.5 µL, 1 mg/mL), sgRNA (25 µL, 0.5 nmol/mL) and 10× lysis buffer (10 µL) were incubated at 37℃ for 10 min to form the Cas14a-sgRNA complex. Then the SDA product (20 µL) corresponding to the target at different concentrations was added, and supplemented with double-distilled water until the total volume was 100 µL. The CdS-Au-UCNPs/ITO working electrode was soaked in the above solution or the above solution was added dropwise onto the electrode surface, and placed at 37℃ for 1 h.

**V. PEC detection**:

A three-electrode system was adopted, with CdS-Au-UCNPs/ITO as the working electrode, Ag/AgCl electrode as the reference electrode, and Pt sheet electrode as the auxiliary electrode. With 980 nm light as the excitation light source, and 0.1 M anhydrous PBS (pH 7.41) and 0.01 M AA as the electrolyte, PEC detection was performed using the current-time curve method under bias voltage of 0 V. Before use for detection, it was necessary to fill the electrolyte with high-purity nitrogen for 20 min. After the detection procedure was started, the nitrogen was maintained above the electrolyte to keep reactants in a nitrogen environment.

### Fluorescence detection methods

Magnetic bead recognition, SDA isothermal amplification and cleavage steps were same as “PEC detection methods”. The above tubes were transferred to a micro-fluorescence cuvette, and water added until the total volume was 150 µL. A fluorescence spectrophotometer was then used to detect the peak value at 482 nm at the excitation wavelength of 980 nm and current of 0.5 A.

### Pretreatment of actual samples

Oats: The oats were purchased from a local supermarket, and the sample was prepared mainly according to the work of M Pascale [31]. 5 g of ground oats were extracted with 25 mL of methanol/water (70:30, v/v) for 50 min at room temperature, followed by centrifugation at 4,000 rpm for 10 min and filtering through filter paper. The supernatant was harvested and diluted 1:5 with deionized water. The diluent was used for sample detection.

Serum: Pretreatment was conducted according to the literature [32]. The human serum collected from volunteers in a hospital in Tianjin was diluted 30-fold with TM buffer for later use.

**Statistical analysis.** All experiments and assays were repeated at least three times. The data in figures were expressed as mean ± standard deviation (SD). The Origin 8 were used for data analysis.

## Results and discussion

### Principle of detection by the UCNPs-Cas14a-based PEC sensor

**Fig. 1:**
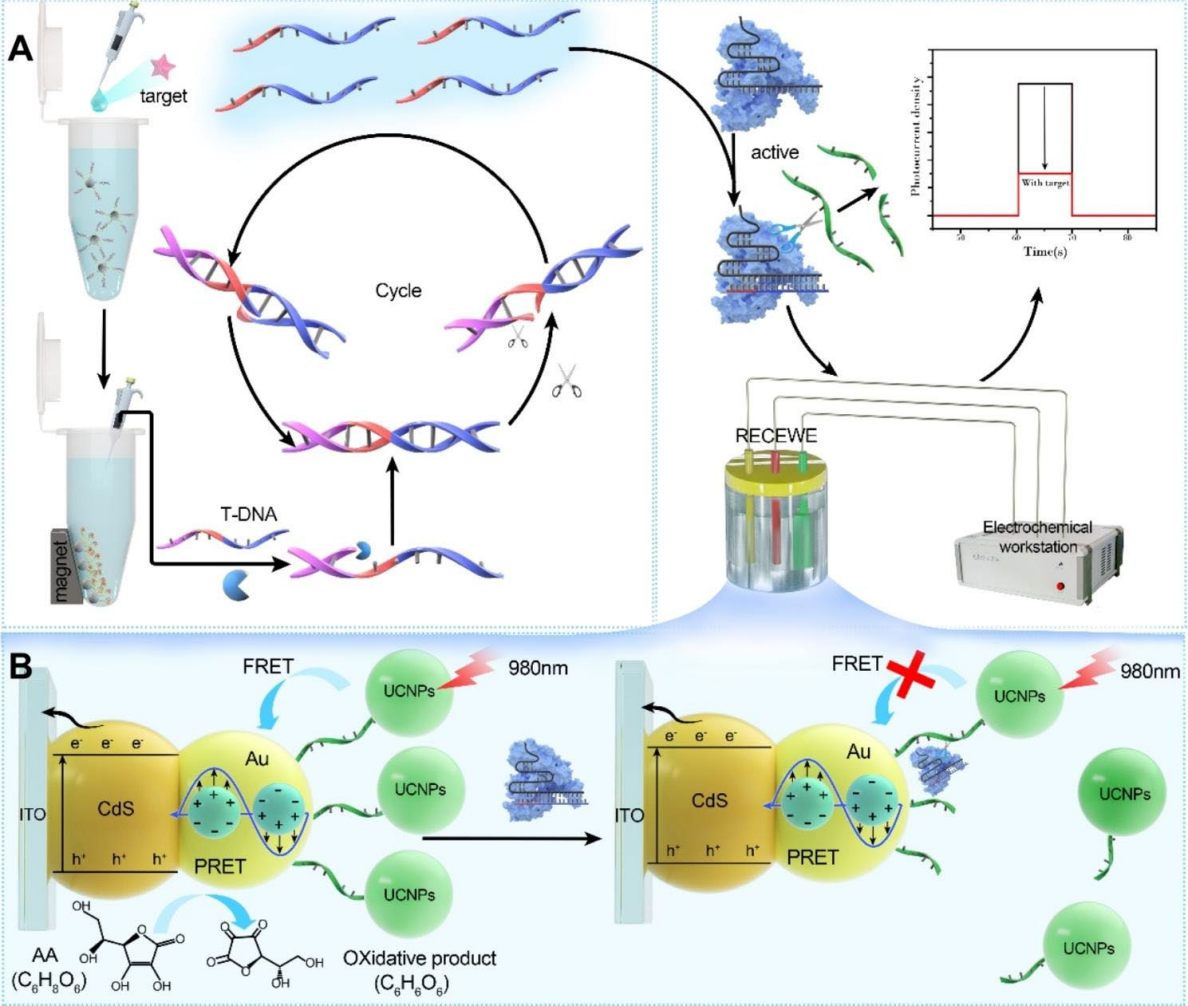
Detection schematic illustration. **A**) Detection principle of UCNPs-Cas14a based PEC. **B**) Schematic diagram of electrical signal output

A simple signal conversion method was introduced by using UCNPs-Cas14a-based PEC, in order to extend the types of analytes that can be detected by CRISPR-based sensors. At the same time, the sensor achieved photoelectric signal output through the ingenious combination of CRISPR and nanomaterials, thereby further improving the sensitivity of detection. The working principle of the UCNPs-Cas14a-based PEC sensor is mainly divided into three stages (Fig. 1): 1) signal identification and conversion depending on the magnetic bead ATP-cDNA complex; 2) a large number of target chains is obtained through SDA for signal amplification; 3) signal output impacts Cas14a-sgDNA the cleavage activity of which can be activated by these target DNA. The PEC workstation consists of the working electrode UCNPs-ssDNA-CdS@Au-modified ITO, the counter electrode which is a platinum sheet, and the reference electrode which is Ag/AgCl. A non-specific ssDNA reporter is incorporated into the PEC workstation, which possesses UCNPs for absorbing NIR light (980 nm) and transferring this energy to CdS@Au/ITO; the sulfhydryl group tethered to the surface of the sensor thereby results in the generation of electrical signals (Fig. 1B). In the presence of the target, cDNA dissociates from the aptamer-binding region because the aptamer on the magnetic beads binds to the target. Free cDNA collected by magnetic separation induces SDA (whereby cDNA and template strand bind to and polymerize along the 5’-3’ direction with the help of polymerase, after which Nt.BsmAI recognizes and cleaves one of the double strands to form a gap, so that KF can enter and polymerize to complete the double strands, and the cleaved fragments are replaced, continuing in this way for several cycles). This process produces a large amount of ssDNA, which triggers the cleavage activity of the Cas14a-sgRNA complex, cleaving UCNPs from the surface of the CdS@Au/ITO electrode. With increasing distance between UCNPs and CdS@Au/ITO, it becomes increasingly difficult to transfer the NIR light absorbed by UCNPs to CdS@Au/ITO, so the electrical signals become weaker. In the absence of the target, the cleavage activity of Cas14a-sgRNA is silenced, so the structure of UCNPs-ssDNA-CdS@Au/ITO remains intact. On the basis of this strategy, a universal UCNPs-Cas14a-based PEC sensor was developed for signal transduction in a simple and efficient manner, as reported in this study.

### Feasibility and characterization of the UCNPs-Cas14a-based PEC sensor

**Fig. 2:**
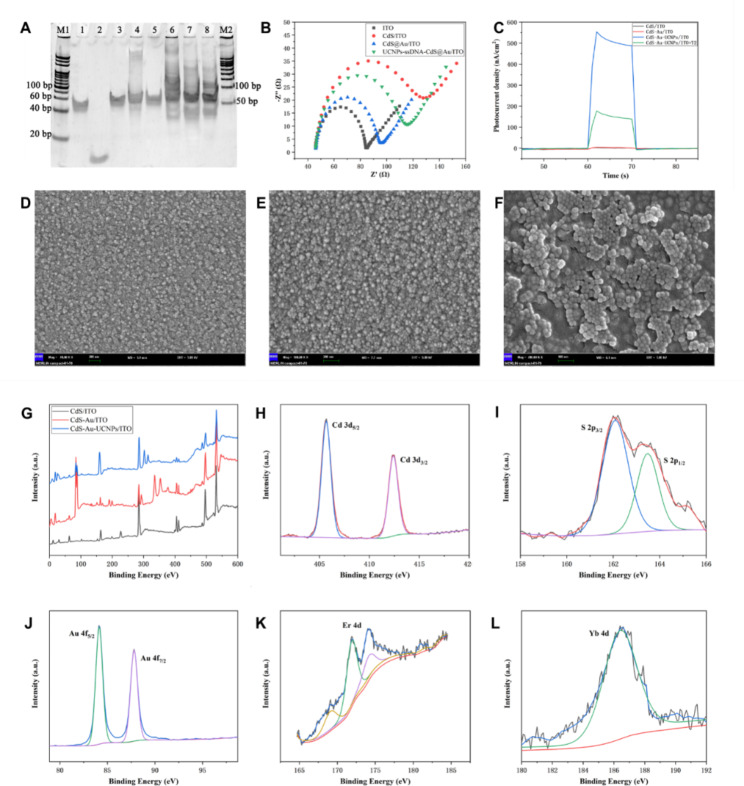
Feasibility and electrode characterization of UCNPs-Cas14a based PEC. **A**) PAGE electropherograms to verify complementary strand triggered SDA of T2 aptamers (M1: 20 bp marker; 1: T-DNA; 2: cDNA; 3: cDNA + T-DNA; 4: cDNA + T-DNA + Nt; 6/7/8: 0.5/2/5 µM cDNA + T-DNA + KF + Nt; M2: 50 bp marker). DNA + KF; 5: cDNA + T-DNA + Nt; 6/7/8: 0.5/2/5 µM cDNA + T-DNA + KF + Nt; M2: 50 bp marker). **B**) AC impedance spectrum of the modified electrode [5 Mm Fe(CN)63-/4-(1:1), 0.1 M KCl, 100 KHZ-0.1 HZ]. **C**) Feasibility validation of UCNPs-Cas14a based PEC. **D**) SEM of CdS/ITO. **E**) SEM of CdS@Au/ITO. **F**) SEM of UCNPs-ssDNA-CdS@Au/ITO. **G**) XPS spectra of modified electrodes. XPS spectra of modified electrodes of Cd (**H**), S (**I**), Er (**J**), Au (**K**) and Yb (**M**)

T2 was selected for evaluating the performance of the UCNPs-Cas14a-based PEC sensor for detecting the target of choice. The T-2 toxin is one of the most potent naturally-occurring type A mucorene toxins secreted by fusarium, which is widely present in foods such as corn and wheat [33, 34]. T-2 is toxic in trace amounts, and causes damage to multiple organs such as the liver, brain and reproductive system after entering the human body [35–37]. Therefore, it is of great significance to develop an ultrasensitive and highly-stable strategy for detecting and quantifying T2. The key to the successful detection of T2 by the UCNPs-Cas14a-based PEC sensor is that a large amount of ssDNA obtained after SDA on the complementary strand corresponding to the T2 aptamer can bind to sgRNA, thereby triggering the cleavage activity of Cas14a and causing changes in the photocurrent. First, the complementary strand [38], template strand and corresponding sgRNA sequence were designed based on the T2 aptamer. The effect of cDNA-induced SDA was verified through polyacrylamide gel electrophoresis (PAGE). As shown in Fig. 2A, in the presence of only T-DNA in the system, no new bands were found in Lane 1, indicating that no amplification had occurred. In the presence of both complementary strand and T-DNA but without enzyme (Lane 3/4/5), no new bands were found either, but the original band at 50 bp was shifted slightly upward, confirming pairing between the complementary strand and T-DNA. In the presence of the complementary strand, T-DNA and enzymes, new bands appeared in Lane 6/7/8 at about 30 bp, similar in length to the target ssDNA, indicating that SDA had been successful and the target product generated. Moreover, with increasing cDNA concentration, the color of the new bands became deeper. These results demonstrate that cDNA can specifically induce SDA, and that the amount of ssDNA SDA is directly proportional to the cDNA concentration. In addition, to confirm the ability of ssDNA SDA to activate Cas14a, PEC studies were further conducted. It could be observed under a scanning electron microscope (Fig. 2D, E and F) that the electrode was successfully modified with CdS, Au and UCNPs. Alternating-current impedance spectroscopy is a highly effective method to describe the resistance of surface modified electrodes, and the diameter of the semicircle in the impedance spectrum corresponds to the value of resistance (Ret) of electron transfer. By comparing the curves in Fig. 2B, it can be seen that after modification with Au-NPs, the ITO Ret slightly declined, which was related to the excellent conductivity of Au. After modification with CdS QDs, the ITO Ret greatly increased, the reason being that the quantum dot (QD) itself is a semiconductor with poor conductivity. After deposition of Au nanoparticles on the surface of CdS QDs, the modified electrode Ret declined but was still higher than that of the electrode modified by Au alone. Moreover, the UCNPs-ssDNA-CdS@Au/ITO electrode Ret was higher than that of CdS@Au/ITO, because the conductivity of UCNPs is also poor. However, its Ret was lower than that of CdS/ITO, which may also be attributed to the excellent conductivity of Au. In the absence of T2, the photoelectric signal observed was the strongest, because Cas14a is not activated and upconversion occurs close to the electrode surface (Fig. 2C). In the presence of T2, the photoelectric signal became weaker, inversely proportional to the T2 concentration. This indicates that the cleavage activity of Cas14a had been triggered, so that the upconversion occurs far away, thus hindering energy transfer. The full XPS spectra of the modified electrode in each step are shown in Fig. 2G-M. Additional characterization of the working electrode UCNPs-ssDNA-CdS@Au/ITO and photoelectric signal conversion process analysis was shown in the supporting information.

### Optimization of the detection performance of the UCNPs-Cas14a-based PEC sensor

**Fig. 3:**
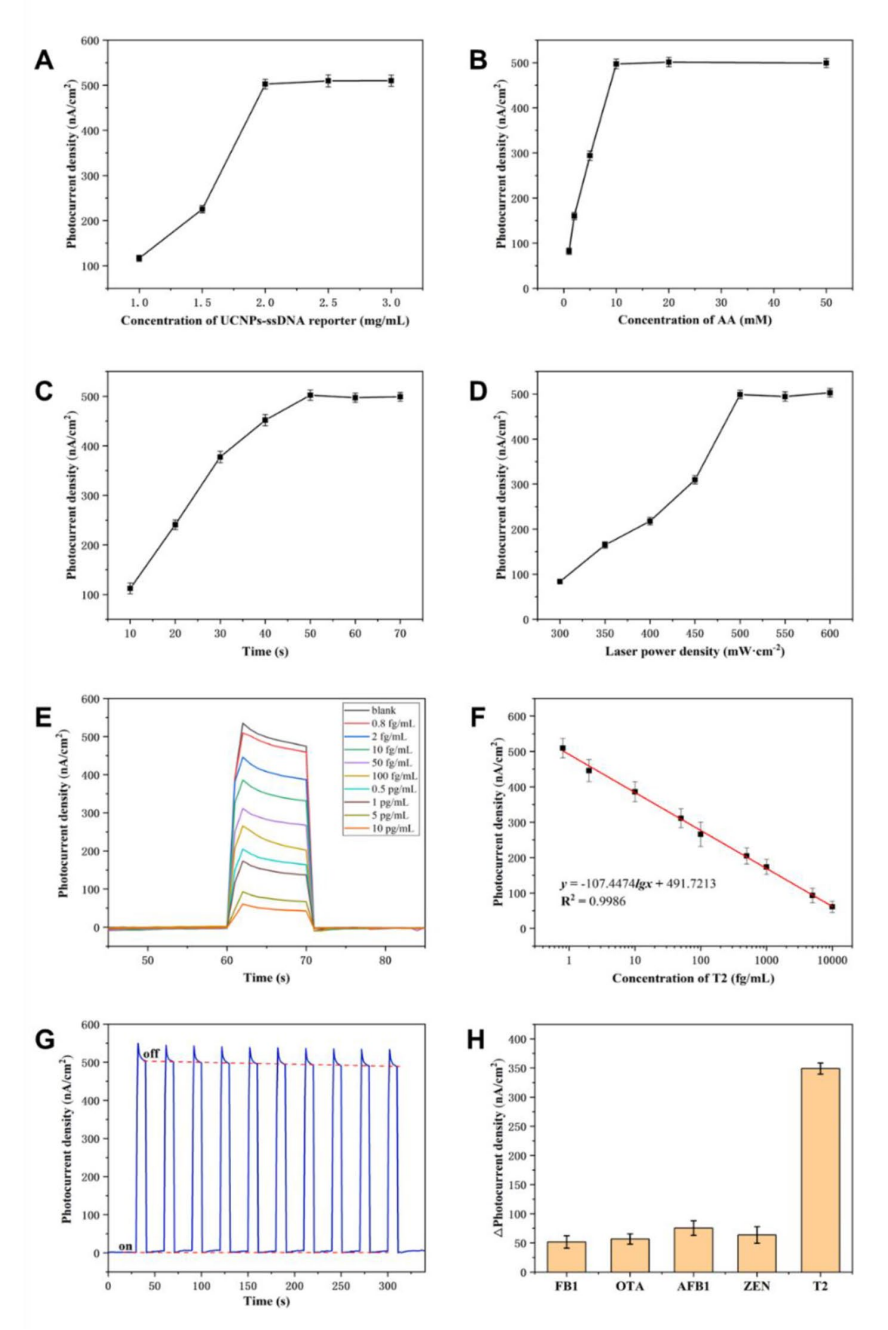
UCNPs-Cas14a based PEC assay for T2. **A**) Optimization of UCNPs-ssDNA concentration. **B**) Optimization of AA concentration. **C**) Optimization of reaction time. **D**) Optimization of laser intensity. **E**) I-T bar curves corresponding to different concentrations of T2. **F**) Standard curves of UCNPs-Cas14a based PEC for T2 detection. **G**) Stability of UCNPs-Cas14a based PEC sensing. **H**) Specificity of UCNPs-Cas14a based PEC sensing

The cleavage efficiency of Cas14a protein and conversion efficiency of photoelectric signals are extremely important factors influencing the detection performance of UCNPs-Cas14a-based PEC sensors. In order to achieve an efficient surface chemistry-based trans-cleavage, accessibility of Cas14a to the nonspecific ssDNA is crucial. Therefore, the influence of the density of the UCNPs-ssDNA reporter on the electrode surface on the changes in photoelectric signals before and after cleavage was optimized in this study. As can be seen from Fig. 3A, the ideal concentration of the UCNPs-ssDNA reporter was 2 mg/mL, creating enough space for cleavage of Cas14a on the electrode surface. High-concentration ssDNA reporter molecules will reduce the signal change value, because such high surface density will lead to a steric-hindrance effect, thereby limiting cleavage activity. Moreover, the efficiency of conversion of photoelectric signals is affected by the AA concentration in the electrolyte, by the cleavage time and by the intensity of irradiation by the laser. As shown in Fig. 3B, C and D, with increasing AA concentration, cleavage time and laser intensity, the photocurrent was gradually enhanced until a plateau was reached. Considering cost savings and guaranteeing photoelectric conversion efficiency, the optimal detection conditions were determined. These were as follows: AA concentration 10 mM, cleavage time 50 min and laser intensity 500 mW·cm^− 2^.

### T2 detection using the PEC sensor

To determine whether the UCNPs-Cas14a-based PEC sensor can detect T2 in a quantitative manner, the photocurrent corresponding to different concentrations of T2 was first measured in a separate device. Signal identification, conversion and amplification experiments were conducted in an EP tube, and the photocurrent was measured in a detection cell fabricated in-house. It was found that the photocurrent was enhanced with increasing T2 concentration from 0.8 fg/mL to 10,000 fg/mL (Fig. 3E). There was a good linear relationship between photocurrent density and the logarithm of T2 concentration (Fig. 3F). The regression equation was *y* = -107.4474lg*x* + 491.7213 (fg/mL, R^2^ = 0.9958, n = 8). According to m+-3σ (where σ is standard deviation of the blank, and m is the mean of 10 blank samples), the limit of detection was estimated to be 0.5128 fg/mL. The I-T columnar curve was plotted under a periodic cycle of light “on” and “off” to investigate the stability of UCNPs-ssDNA-CdS@Au/ITO in detecting T2 (Fig. 3G). The results showed that the relative standard deviation (RSD) of the photoelectric response value to T2 was 0.68% under 10 cycles of light “on” and “off”, indicating that the electrode had good stability.

**Fig. 4:**
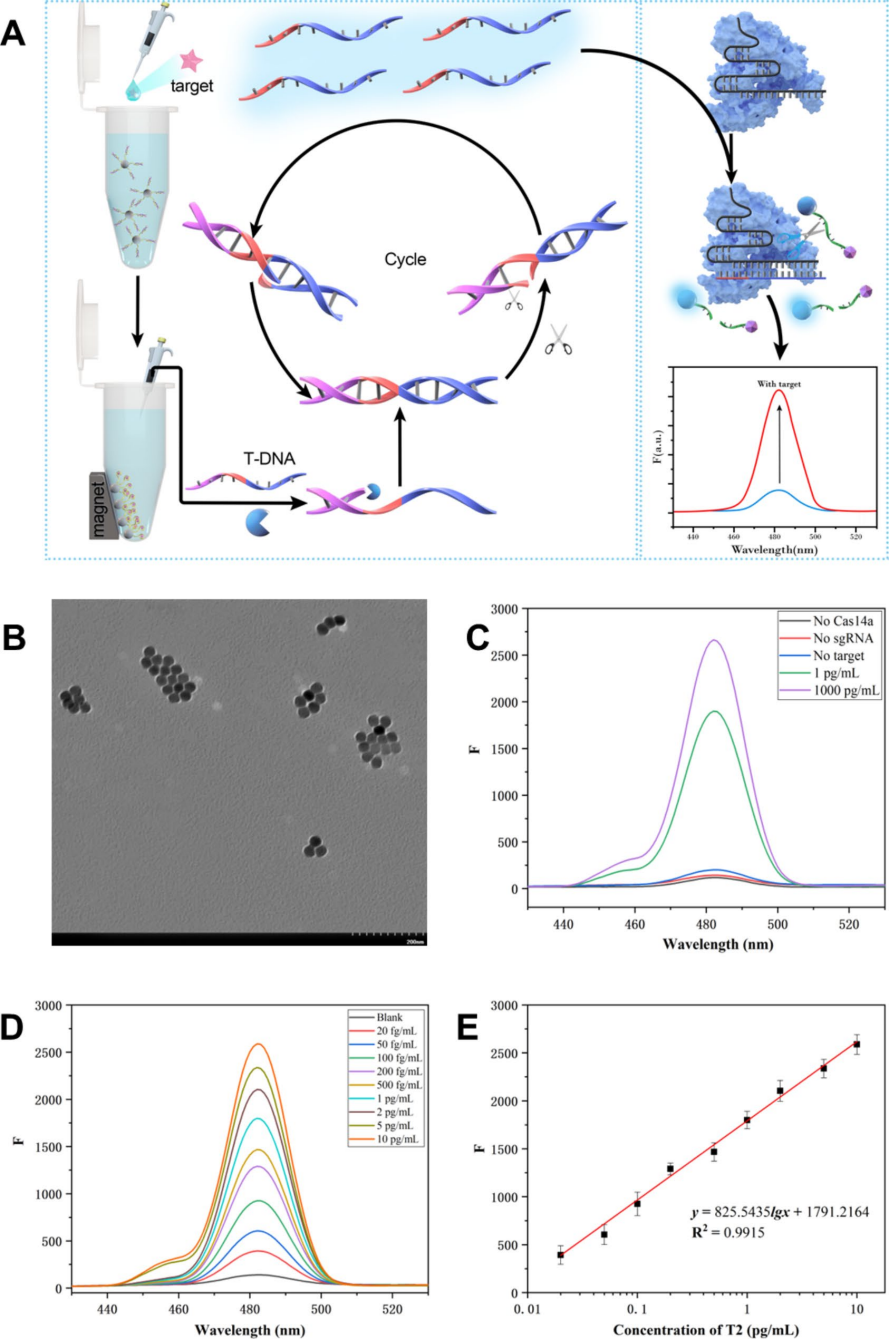
UCNPs-Cas14a based FL detection of T2. **A**) Schematic illustration of UCNPs-Cas14a based FL detection. **B**) TEM of UCNPs, **C**) Feasibility of fluorescence detection. **D**) Fluorescence spectra corresponding to different concentrations of T2. **E**) Standard curve of UCNPs-Cas14a based FL for T2 detection

At the same time, a Cas14a-based upconversion fluorescence (UCNPs-Cas14a-based FL) sensor was also constructed to detect T2 as a comparison (Fig. 4A). UCNPs were characterized by TEM, showing that they had a spherical shape with a particle size of about 30 nm (Fig. 4B). The results shown in Fig. 4C indicated that fluorescence was significantly generated only in the presence of the target. The optimization was shown in Figure S4. The signal identification, conversion and amplification properties of the UCNPs-Cas14a-based FL sensor were the same as those for the UCNPs-Cas14a-based PEC sensor, but with a fluorescence signal output. In other words, the combination of SDA product ssDNA and Cas14a-sgRNA complex can activate the ssDNA of the UCNPs-ssDNA-D probe in the Cas14a trans-cleavage system. As the distance between UCNPs and Dabcyl became larger, the fluorescence of UCNPs at 482 nm recovered. It could be seen from Fig. 4D and E that the T2 concentration was directly proportional to the fluorescence intensity. When the T2 concentration was within 0.02–10 pg/mL, the linear equation was *y* = 825.5435lg*x* + 1791.2164 (pg/mL, R^2^ = 0.9915, n = 8, LOD = 0.0133 pg/mL). Compared with UCNPs-Cas14a-based FL sensors, the sensitivity of the UCNPs-Cas14a-based PEC sensor was improved about 25-fold, while its stability was not inferior. Therefore, it is concluded that the high sensitivity of UCNPs-Cas14a-based PEC sensors benefits from the triple amplification of SDA, Cas14a trans-cleavage feature and photoelectric signals.

### Evaluation of detection performance of PEC sensor in terms of specificity and measurement of T2 in oats

In view of the detection performance of the UCNPs-Cas14a-based PEC sensor under experimental conditions, it is crucial to confirm its specificity and practical application for target detection. To this end, the specificity of the UCNPs-Cas14a-based PEC sensor was tested using analogues of the T2 toxin, such as zearalenone (ZEN), ochratoxin A (OTA), fumonisin (FB1) and aflatoxin B1 (AFB1). As shown in Fig. 3H, even high concentrations of these analogues barely caused any changes in the generated photocurrent compared with the background/blank signals. In contrast, T2 toxin at a concentration 100 times lower than the analogues still caused clearly measurable changes in the photocurrent. Therefore, the UCNPs-Cas14a-based PEC sensor can be used for highly specific T2-targeted detection.

Next, oats were selected as a representative of actual real-world samples and T2 detected by the UCNPs-Cas14a-based PEC sensor was assessed. Method comparison was also performed with a commercial kit (limit of detection 15 pg/mL). The ELISA kit results were in good agreement with the UCNPs-Cas14a-based PEC sensor. The resulting analysis in Table S1 revealed that the standard-addition recovery rate of T2 in oats was 84.49–118.30%, and its RSD was 1.4–2.9%, suggesting that the UCNPs-Cas14a-based PEC sensor can serve as an optional method for quantitative detection of T2 in oats.

### Detection of protein tyrosine kinase 7 (PTK7) used PEC sensor

**Fig. 5:**
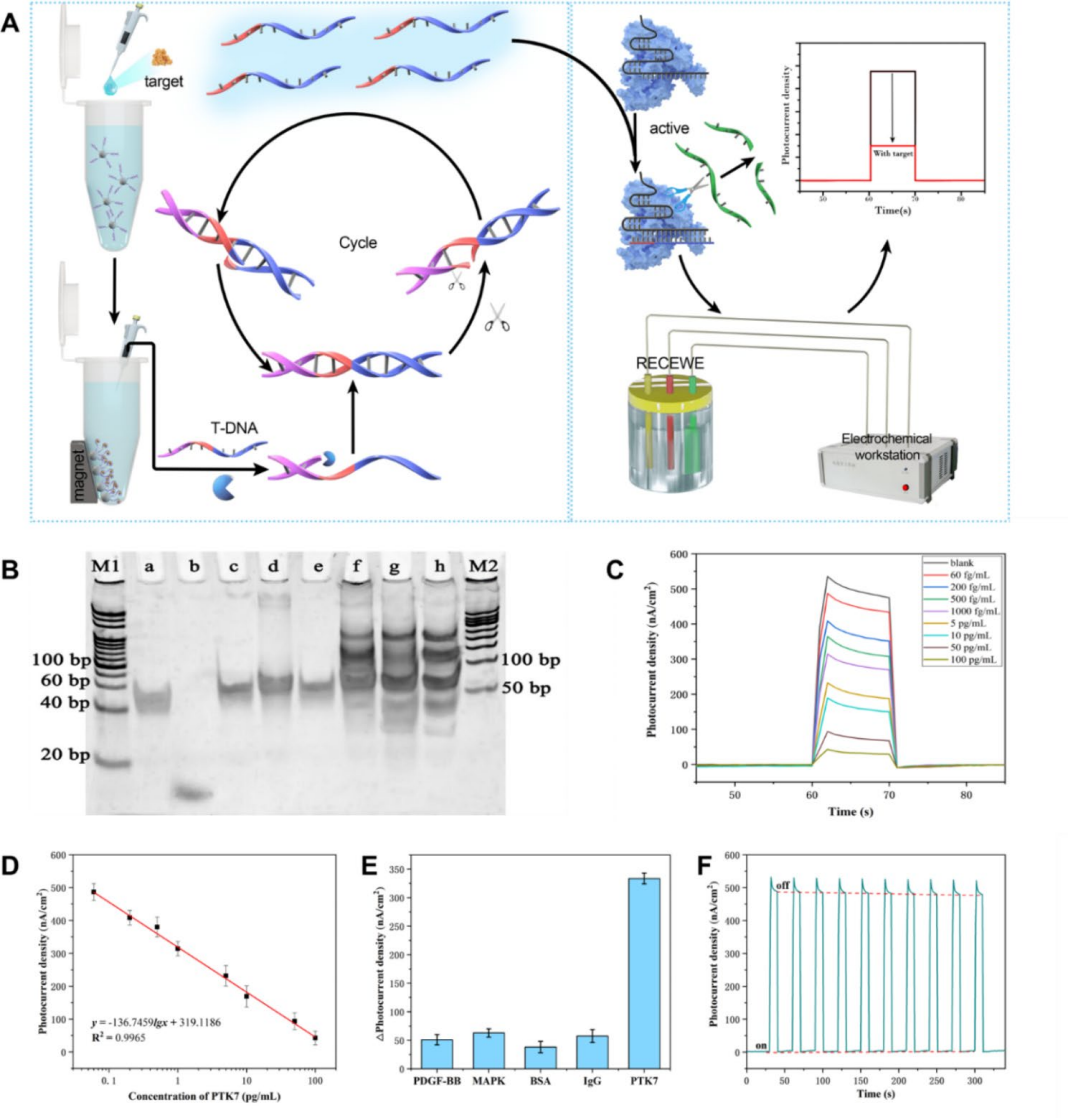
UCNPs-Cas14a based PEC for PTK7 detection. **A**) Schematic illustration of UCNPs-Cas14a based PEC for PTK7 detection. **B**) PAGE electropherogram verifying the SDA triggered by the complementary DNA of PTK7 aptamer (M1: 20 bp marker; a: T-DNA; b: cDNA; c: cDNA + T-DNA; d: cDNA + T-DNA + KF; e: cDNA + T-DNA + Nt; f/g/h: 0.5/2/5 µM cDNA + T-DNA + KF + Nt; M2: 50 bp marker). **C**) I-T bar curves corresponding to different concentrations of PTK7. **D**) Standard curve of UCNPs-Cas14a based PEC for the detection of T2. **E**) Specificity of UCNPs-Cas14a based PEC sensing. **F**) Stability of UCNPs-Cas14a based PEC sensing to detect PTK7

To investigate the general applicability of the UCNPs-Cas14a-based PEC sensor, its ability to detect protein targets was explored. PTK7 is a kinase that catalyzes the transfer of γ-phosphate from ATP to protein tyrosine residues. It plays an important role in cell growth, proliferation and differentiation [39, 40]. Most PTKs discovered so far are oncogene products of oncogenic RNA viruses, and they can also be produced by proto-oncogenes in vertebrates [41]. Therefore, they are important tumor markers in the clinic [42]. Here, PTK7 was used as the target, and the aptamer served as the recognition element of PTK7. The principle for its detection is shown in Fig. 5A. In the presence of PTK7, the corresponding cDNA was dissociated and could be collected through magnetic separation. cDNA could induce SDA to obtain a large amount of ssDNA, thereby triggering the cleavage activity of the Cas14a-sgRNA complex, and leading to cleavage of UCNPs from the surface of the CdS@Au/ITO electrode. As a result, energy transfer was hindered, weakening the electrical signals. The gel electrophoretogram (Fig. 5B) showed that the complementary strand could successfully induce SDA, that is, new bands appeared at about 30 bp in the Lane f/g/h. There were no new bands (Lane a/b/c/d/e) when either the T-DNA, or enzyme, or cDNA was absent.

Accordingly, detection of PTK7 using the UCNPs-Cas14a-based PEC sensor was assessed. As shown in Fig. 5C and D, there was a good linear relationship between photocurrent density and the logarithm of PTK7 concentration when the latter was within the range 0.06–100 pg/mL. The regression equation was *y* = -136.7459lg*x* + 319.1186 (pg/mL, R^2^ = 0.9977, n = 8). The calculated limit of detection was 0.03783 pg/mL.

The UCNPs-Cas14a-based PEC sensor showed better selectivity for PTK7 at a concentration of 10 pg/mL than for the other competing proteins tested (platelet-derived growth factor-BB, mitogen-activated protein kinase, BSA and IgG) (Fig. 5E). It can be seen that the aptamer employed has excellent specificity in the sensor. The evaluation of stability was shown in Fig. 5F. All baseline and photocurrent responses (n = 10) were relatively stable in “on” and “off” states, respectively.

Furthermore, the ability of the UCNPs-Cas14a-based PEC sensor to detect PTK7 in human serum samples was also tested. The results showed (Table S2) that the standard-addition recovery rate in human serum was 83.34–118.40%, and its RSD was less than 4%, confirming the reliability of detection by this UCNPs-Cas14a-based PEC sensor. Moreover, the aptamer is a universal recognition element for proteins and small molecules. Therefore, the UCNPs-Cas14a-based PEC sensor can be applied to a variety of analytes.

## Conclusion

In this study, a new strategy for the construction of a universal PEC sensor was reported, i.e., the cleavage activity of CRISPR-Cas14a (UCNPs-Cas14a based-PEC sensor) was manifested by altered photoelectric signals. Unlike other proteins in the Cas family, Cas14a has a low molecular weight and is more specific in identifying ssDNA. Electrical signals are more cost-effective to measure with portable equipment than fluorescence signals. In this study, the sensitivity of detection by the PEC sensor was improved by using an NIR light source and SDA, while its stability was enhanced by the precise target identification properties of CRISPR-Cas14a. The UCNPs-Cas14a-based PEC sensor converts aptamer recognition events into detectable PEC signals through Cas14a-sgRNA and UCNPs-ssDNA-CdS@Au ITO electrodes. T2 and PTK7 can be quantitatively detected by this sensor in a simple and rapid manner, and it can also be used for the analysis of actual samples of foodstuffs and human serum in the examples presented here. In addition, as a universal sensor, the UCNPs-Cas14a-based PEC expands the range of applications of the gene-editing tool CRISPR from the detection of nucleic acids to small molecules and proteins. To summarize, the successful construction of UCNPs-Cas14a-based PEC sensors has tremendous potential for enriching the type of output signal of the CRISPR-CAS system and facilitating its application in more fields, such as toxicological analysis and clinical diagnosis.

### Electronic supplementary material

Below is the link to the electronic supplementary material.

Supplementary Material 1

## Data Availability

The data that support the findings of this study are available from the corresponding author upon reasonable request.
