# Supplementary material for: A universal CRISPR-Cas14a responsive triple-sensitized upconversion photoelectrochemical sensor
Source: J Nanobiotechnology. 2023 Oct 26;21:389. doi: 10.1186/s12951-023-02163-z (PMC10601294; doi:10.1186/s12951-023-02163-z)
Supplement: Supplementary file 1 — Supplementary Material 1 [file 12951_2023_2163_MOESM1_ESM.docx]

**Supplementary Material**

**A universal CRISPR-Cas14a responsive triple-sensitized upconversion photoelectrochemical sensor**

Yu Wang^1^, Yuan Peng^1^, Huanying Zhou^1^ and Zhixian Gao^1*^

1 Tianjin Key Laboratory of Risk Assessment and Control Technology for Environment and Food Safety, Tianjin Institute of Environmental and Operational Medicine, Tianjin 300050, P.R. China. E-mail: gaozhx@163.com; Tel.: +86 22 84655403; fax: +86 22 84655403.

**Experimental Section**

**Reagents and instruments**

The fluorescence spectrophotometer F9300 and electrochemical workstation used in this work were purchased from Shanghai Prism Technology Co., Ltd (China) and Ivium Technologies (Eindhoven, The Netherlands), respectively. Indium tin oxide (ITO) transparent conductive glass (1.1 mm thick, square resistance ≤100 ohms, Suzhou Itanetron Electronics Co.) Cadmium chloride (AR), sodium thiosulfate (AR), CH3CSNH2, sodium hexametaphosphate, cysteine, and hydrochloric acid (concentration 0.1 M) were purchased from McLean Biochemical Technology (Shanghai, China); HAuCl_4_-4H_2_O (AR) was bought from Sigma-Aldrich, Inc. (St. Louis, MO, USA). Aminated upconversion nanoparticles were purchased from Xi'an Ruixi Biotechnology (China). T2 toxin and PTK-7 were purchased from Sigma-Aldrich, Inc. (St. Louis, MO, USA) and R&D Systems (Minnesota, USA), respectively. Streptavidinized magnetic beads were purchased from Beaver (Suzhou, China). Cas14a1 was purchased from Shanghai Nearshore Protein Company (China). klenow Fragment (3′→5′exo-), NtBsmAI, dNTP were purchased from New England BioLabs Inc (Ipswich, UK).

DNA sequences were synthesized by Biotech Biotechnology Co., and RNA was synthesized by Suzhou Hongxun Biologicals Inc.

**Table S1. Sequences of DNA and RNA used in the experiments**

| Name | DNA sequence 5’-3’ |
| --- | --- |
| T2-APT | bio-CAGCTCAGAAGCTTGATCCTGTATATCAAGCATCGCGTGTTTA  CACATGCGAGAGGTGAAGACTCGAAGTCGTGCATCTG |
| PTK7-APT | bio-ATCTAACTGCTGCGCCGCCGGGAAAATACTGTACGGTTAGA |
| T2-cDNA | ACCTCTCGCATGTGT |
| PTK7-cDNA | CAGTATTTTCCCGTC |
| T2-T-DNA | CATTAAAAATACCGAACGAACCACCAGCAGAAGATAAAACAGAGACACATGCGAGAGGT |
| PTK7-T-DNA | CATTAAAAATACCGAACGAACCACCAGCAGAAGATAAAACAGAGACGGGAAAATACTG |
| FL-DNA_Reporter_ | NH_2_-TTTTTTTTTT-Dabcyl |
| PEC-DNA_Reporter_ | SH-C3-TTTTTTTT-C6-NH_2_ |
| sgRNA | CUUCACUGAUAAAGUGGAGAACCGCUUCACCAAAAGCUGUCCCUUAGGGGAUUAGAACUUGAGUGAAGGUGGGCUGCUUGCAUCAGCCUAAUGUCGAGAAGUGCUUUCUUCGGAAAGUAACCCUCGAAACAAAUUCAUUUUUCCUCUCCAAUUCUGCACAAGAAAGUUGCAGAACCCGAAUAGACGAAUGAAGGAAUGCAACUACCGAACGAACCACCAGCAGAAGA |

**Preparation of magnetic probes**

Aptamers were modified on magnetic beads according to the instructions provided by the manufacturer) (BeaverBeads™ Streptavidin). The size of the magnetic beads used in the experiment was approximately 300 nm. First, 100 μL of magnetic beads was placed into a new centrifuge tube, which was then placed in a magnetic stirrer for 1 min. The supernatant was then removed and the operation called as the ‘magnetic separation’ in the next stage. The magnetic beads were then washed three times with 1 mL of buffer I 10 mM of Tris-HCl (pH=7.5), 1 mM of EDTA, 1 M of NaCl, and 0.01-0.1% of Tween-20). Next, a total of 500 μL of biotinylated aptamers (final DNA concentration: 2 nmol/mL) was added to the ‘magnetic separation’ which was then rotated and mixed for 30 min at room temperature. After magnetic separation, the magnetic beads were washed three times, centrifuged as before, and resuspended for later use.

**SDA method**

(1) TemDNA (1 μmol/L, 1 μL) and cDNA (5.5 μL) corresponding to the target at different concentrations were heated in a hot water bath at 95°C for 5 min, and then slowly cooled to room temperature for 2 h.

(2) 1 μL of CutSmart (10×) buffer, 1.5 μL of dNTP (10 mmol/L), and 0.5 μL of Klenow Fragment (3'-5' exo-) (5,000 U/mL) were added into the mixture from (1), and mixed evenly for reaction at 37℃ for 15 min.

(3) 1 μL of Nt.BsmAI enzyme (10,000 U/mL) was added into the mixture from (2), and ultrapure water was added until the total volume was 20 μL. Solutions were mixed to homogeneity and incubated at 37°C for 3 h. Finally, the mixture was heated in a hot water bath at 80℃ for 10 min to inactivate the enzyme.

(4) The amplification products were verified through 12% PAGE.

**CdS synthesis method**

(1) 10.0 mL of CdCl_2_ (1.0×10^-2^ mol/L) solution and 10.0 mL of CH_3_CSNH_2_ (1.0×10^-2^ mol/L) solution were added into a 50 mL triangular flask, then 1.00 mL of sodium hexametaphosphate solution (0.10 mol/L) was added dropwise as the stabilizer, and they were mixed fully.

(2) The pH was adjusted to 10.50 with NaOH solution (0.10 mol/L). The reaction mixture was stirred for about 35 min while adding NaOH solution, resulting in the formation of a light yellow CdS nanosol, at which time the pH of solution had decreased to 9.30.

(3) The pH of the CdS nanosol prepared was then adjusted to 7.66, after which a certain amount of cysteine solution at the same pH was added, the total volume was adjusted to 5.0 mL, and the mixture was left to stand for 70 min.

**Connection of upconversion particles and DNA**

(1) 50% glutaraldehyde was diluted with buffer to 2.5%.

(2) Upconversion particles (UCP) (1 mg/mL, 5 mL) and 2.5% glutaraldehyde (10 mL) were mixed allowed to react at room temperature for 1 h.

(3) After centrifugation, the supernatant was discarded, and the material was washed twice with buffer and resuspended at 1.5 mg/mL. Then 100 μL of UCP suspension and cleaved DNA (100 μL, 200 nM) were taken and shaken to facilitate the reaction at a temperature of 25℃ for 3 h. Finally, UCP-D was obtained.

**Results and Discussion**

**Electrode principle**


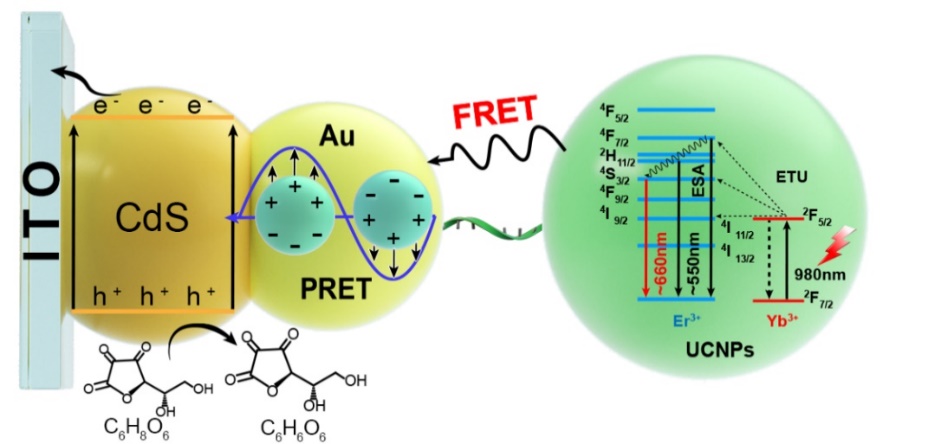


**Figure S1. Schematic** **illustration of energy conversion process of UCNPs-ssDNA-CdS@Au/ITO electrode**

**Electrode characterization diagram, to explain the energy transfer**

As shown in the Figure S2, UCNPs (A) and CdS-QDs (B) are spherical in shape, and have a uniform particle size of 30 nm and 3 nm, respectively. Modification of the working electrode was characterized using fluorescence and ultraviolet. Yb^3+^ as a sensitizer absorbed the incident light from the 980 nm laser, while Er^3+^ as an activator generated multiple emission wavelengths corresponding to the luminescence peaks at 660 nm and 550 nm, respectively. Au absorbed the light emitted by UCNPs through FRET, so the light intensity greatly declined (Figure S2C). The ultraviolet-visible absorption spectra of the modified electrode in each step are shown in Figure S2D. The broad absorption peak of CdS-QDs appeared at 410 nm, indicating that CdS was successfully deposited on the electrode. The peak absorption of Au nanoparticles alone was at 540 nm, while that of Au in CdS-Au/ITO was at 600 nm, the latter being red-shifted relative to the former. This further indicates that Au was deposited on the surface of CdS, consistent with the results from the SEM imaging. In addition, UCNPs had no absorption peak within this wavelength range. The full XPS spectra of the modified electrode in each step are shown in Figure 2G. It can be seen that there were Cd and S elements in CdS/ITO; Cd, S and Au elements in CdS-Au/ITO; and Cd, S, Er, Yb and Au elements in CdS-Au-UCNPs/ITO. The peak shown in Figure 2H appeared at the binding energy of 413.1 eV and 406.6 eV, consistent with the previously-reported peak position of Cd 3d5/2 and Cd 3d3/2, demonstrating that Cd^2+^ is present in the sample. In Figure 2I, the characteristic peaks were located at the binding energy of 162.2 eV and 163.7 eV corresponding to S 2p3/2 and S 2p1/2, respectively, suggesting that the sulfur element in the sample is in the form of S^2-^. The above results indicate that CdS is present in the sample. Additionally, the Au peak (Figure 2J) was located at the binding energy of 87.8 eV (Au 4f7/2) and 84.1 eV (Au 4f5/2); the Er 4d peak (Figure 2K) was located at the binding energy of 174.1 eV and 171.9 eV, and the binding energy peak of S element could be obscured at 168.9 eV because of its low content. The Yb 4d peak (Figure 2L) was located at the binding energy of 186.8 eV. Taken together, the above findings confirm the success of electrode modification.


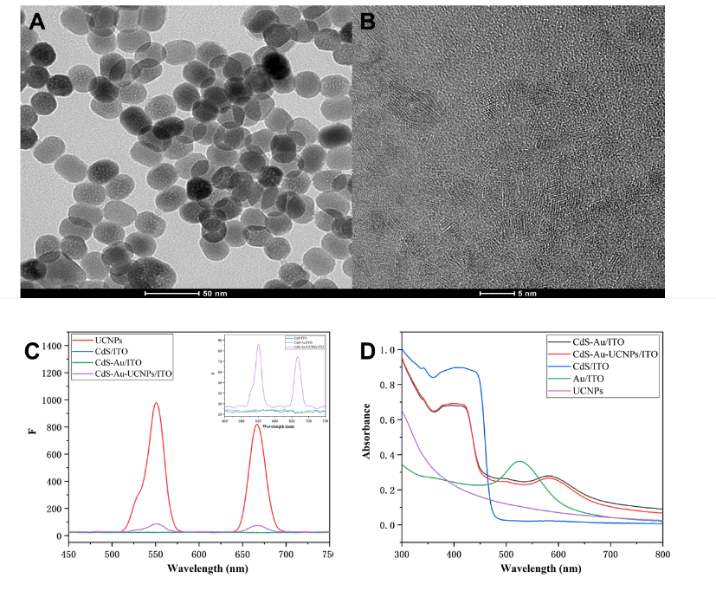


Figure S2. TEM image of UCNPs (A) and CdS-QDs (B), fluorescence spectra (C) and UV absorption spectra (D) of the modified electrodes.

**Loading capacity of magnetic bead-coupled aptamers**

The concentration of nucleic acid in the supernatant before and after the reaction process was measured, so that the amount binding to the magnetic beads could be calculated according to the formula m=(C_b_-C_a_)×V, where m is the amount of nucleic acid modified on the magnetic beads, C_b_ is the concentration of supernatant before the reaction, C_a_ is the concentration of supernatant after the reaction, and V is the reaction volume. The results (Figure S3) showed that the loading capacity of magnetic bead-coupled aptamer was 10.2 μg/mL, that is, the amount of nucleic acid on the magnetic beads in the 500 μL system was 407 pmol.


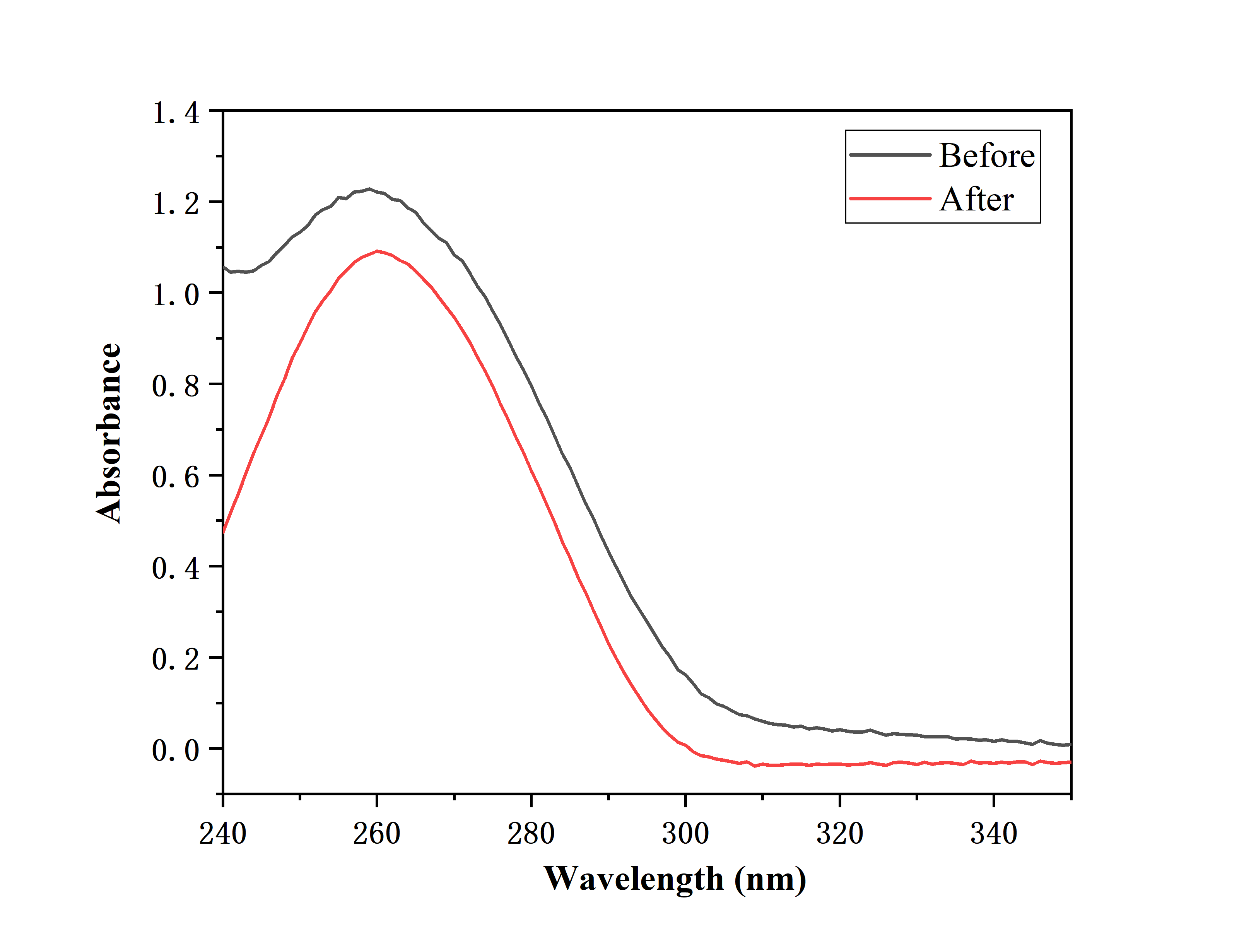


Figure S3. Concentration of aptamer in the supernatant before and after the magnetic bead coupling reaction.

**Optimized results of fluorescence detection**

The amount of UCNPs-ssDNA-D is of great importance for the optimal performance of UCNPs-Cas14a sensors. With increasing concentrations of UCNPs-ssDNA-D (Figure S4), the fluorescence gradually rises and then reaches a plateau. Using this approach, the optimal amount of UCNPs-ssDNA-D was determined to be 4 mg/mL.


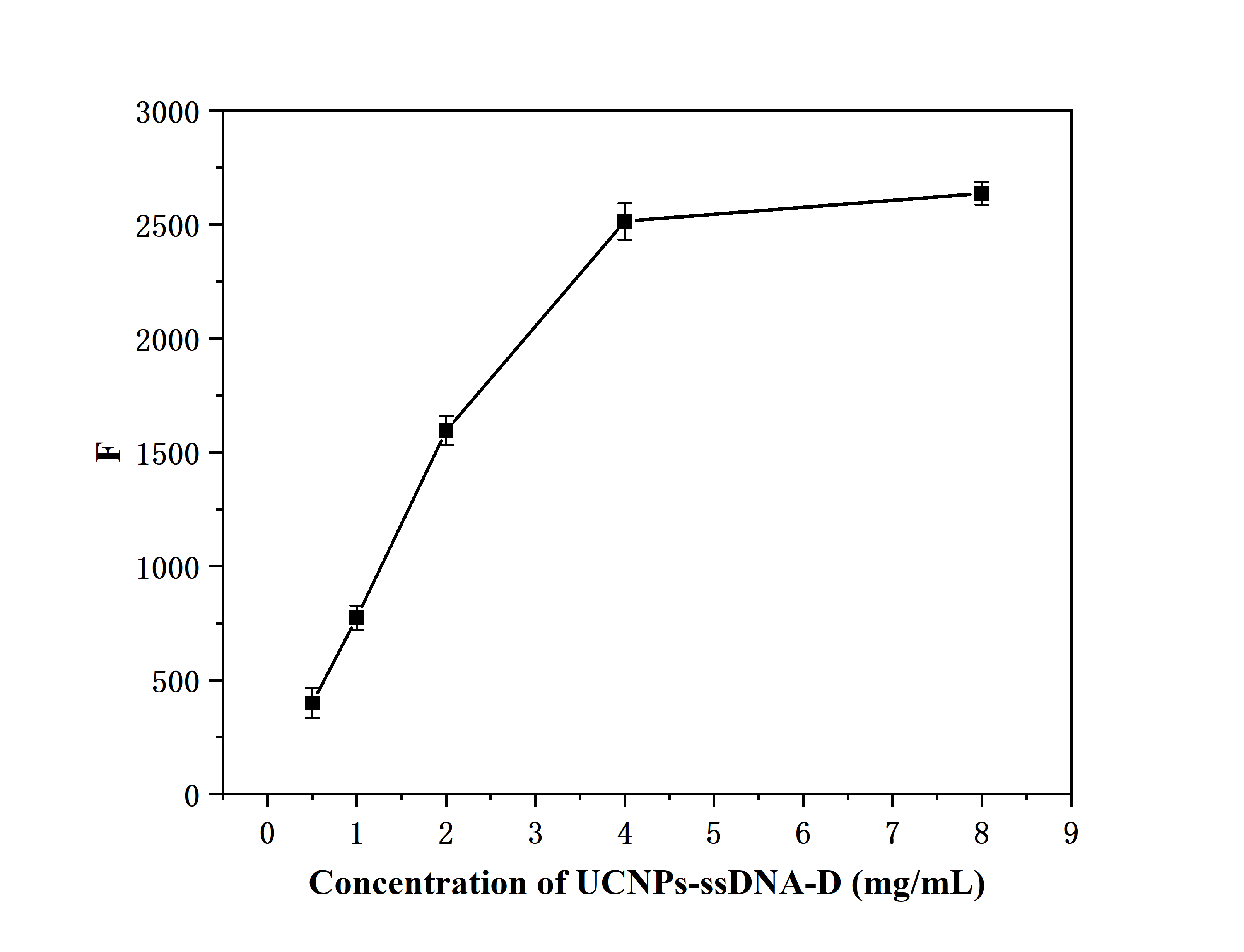


Figure S4. Optimization of the concentration of UCNPs-ssDNA-D.

Table S2. Recovery of three antibiotics at different concentration levels in oats (T2) with UCNPs-Cas14a based PEC (n = 6).

| **Category** | **Samples** | **Spiking levels (fg/mL)** | **Photocurrent density**  **(nA/cm^2^)** | **Amount measured (pg/mL)** | **ELISA kit (pg/mL)** | **Recovery**  **(%)** | **RSD**  **(%)** |
| --- | --- | --- | --- | --- | --- | --- | --- |
| Oats | a | 10 | 377.072 | 11.4815 | - | 114.81 | 1.4 |
|  |  | 100 | 268.895 | 118.304 | 112.865 | 118.30 | 2.6 |
|  |  | 1000 | 171.106 | 954.993 | 987.952 | 95.50 | 2.9 |
|  | b | 10 | 392.140 | 8.449 | - | 84.49 | 1.9 |
|  |  | 100 | 277.685 | 98.177 | 103.886 | 98.18 | 2.0 |
|  |  | 1000 | 175.643 | 874.386 | 902.578 | 87.44 | 2.2 |
|  | c | 10 | 389.297 | 8.979 | - | 89.79 | 2.7 |
|  |  | 100 | 273.585 | 107.193 | 115.773 | 107.19 | 1.8 |
|  |  | 1000 | 171.896 | 947.493 | 899.537 | 94.75 | 1.6 |

Table S3. Recovery of three antibiotics at different concentration levels in human serum samples (PTK7) with UCNPs-Cas14a based PEC (n = 6).

| **Category** | **Samples** | **Spiking levels (pg/mL)** | **Photocurrent density**  **(nA/cm^2^)** | **Amount measured (ng/mL)** | **Recovery**  **(%)** | **RSD**  **(%)** |
| --- | --- | --- | --- | --- | --- | --- |
| Human serum | 1 | 0.2  2 | 404.670 | 0.237 | 118.40 | 2.8 |
|  |  |  | 281.384 | 1.888 | 94.39 | 1.9 |
|  |  | 20 | 140.087 | 20.3817 | 101.91 | 3.2 |
|  | 2 | 0.2 | 417.258 | 0.192 | 95.78 | 3.3 |
|  |  | 2 | 270.325 | 2.274 | 113.71 | 2.5 |
|  |  | 20 | 152.033 | 16.667 | 83.34 | 2.7 |
|  | 3 | 0.2 | 411.412 | 0.211 | 105.69 | 3.0 |
|  |  | 2 | 280.689 | 1.910 | 95.50 | 1.8 |
|  |  | 20 | 145.401 | 18.621 | 93.11 | 3.4 |
